# A terrific journey for which I have great gratitude

**DOI:** 10.4102/sajid.v37i2.400

**Published:** 2022-02-14

**Authors:** Linda-Gail Bekker

**Affiliations:** 1 Desmond Tutu Health Foundation, Cape Town, South Africa; 2 Desmond Tutu HIV Centre, Faculty of Health Sciences, University of Cape Town, Cape Town, South Africa

I am a physician who underwent training in infectious diseases and pursued a PhD programme centred on the host immune response to tuberculosis along with patients harbouring HIV. I am lucky not to be pigeonholed in one area but to have had the opportunity to follow my curiosity and passion – a career gift that I do not take for granted.

I am one of the lucky ones who knew that I wanted to be a doctor from the youngest age. I was raised in Zimbabwe by parents who believed that anything was possible if one set one’s mind to the task at hand. My dad was a wizard with his hands, and despite lacking a tertiary education, he could, in my opinion, fix anything and solve any problem. Growing up on a farm also encouraged curiosity, hard work and a deep sense of appreciation for life, nature and the world in general.

Medical school at the University of Cape Town in the 1980s brought not only excellent training but also rapid consciousness of the sociopolitical issues in South Africa (SA) and, with that, a personal commitment to contribute to the greater good where I could. My internship at McCord Zulu Hospital was followed by 4 years of rural doctoring at the bustling Eshowe Provincial Hospital, KZN. I left medical school with strong intentions to become a geriatrician. Working with the elderly in this small rural community was everything I dreamed of … but in the late 1980s, the HIV epidemic descended on KZN. It was clear that we were losing the battle with multitudes of young people dying despite all our efforts. Frustrated and mortified, the death toll prompted me to gain more skills, feeling that what I had was insufficient. I returned to Groote Schuur and the Department of Medicine to specialise in adult medicine.

A chance encounter with a person who has become a life-long mentor and confidante, Professor Gilla Kaplan, caused the penny to finally drop wherein she suggested that the missing ingredient in my career path was research. With the college exams behind me (PhD followed), I started pursuing my PhD under the supervision of the late Lafras Steyn, wonderful Gary Maartens and Prof. Kaplan. Travelling between Cape Town and Rockefeller University in New York, my world expanded into an international experience. I continued my clinical care work part-time at an HIV clinic run by Prof. Robin Wood at New Somerset Hospital, Cape Town. This unexpectedly but happily led to romance, and Robin and I were married in May 2000, the same year I completed my PhD.

Over the last 21 years, I have been fortunate to develop an academic and a public service career in partnership with Robin. We transferred the HIV Research Unit at New Somerset Hospital and the Infectious Disease Unit at the University of Cape Town Lung Institute to the Institute of Infectious Disease and Molecular Medicine and obtained the agreement of the Archbishop Desmond Tutu to form the Desmond Tutu HIV Foundation. We have since then made research contributions to the prevention and treatment of HIV and TB, focusing in recent years on HIV prevention amongst marginalised and poorly resourced SA populations, specifically adolescents and men who have sex with men. We pushed for antiretroviral therapy (ART) scale-up during the era of AIDS denialism, and we built fleets of mobile clinics called Tutu Testers, research sites, treatment and prevention centres and youth health parks. In 2016, we launched a programme to reach 22 000 young people with sexual health information and services and this year, we plan to bring pre-exposure prophylaxis (PrEP) to 25 000 young adults. From 2016 to 2018, I served as the president of the International AIDS Society, with many opportunities opening as a result. Recently, we joined the fight against coronavirus disease 2019 (COVID-19) by implementing the Sisonke vaccine trial amongst 500 000 healthcare workers in partnership with a colleague and friend, Prof. Glenda Gray.

I learned the power of effective mentors, that generosity in sharing ideas and skills brings many rewards, that there is no substitute for hard work, and surrounding oneself with passionate, creative and committed individuals is the best way to keep one’s own creative and productive batteries fully charged. Finally, while it is vital to focus on locally relevant research to have a tangible impact, there is a wonderful global network of like-minded collaborators – to work locally and collaborate internationally has been most productive!

It has been a terrific journey thus far – that I can truly say was only accomplished through the support of friends and family. Here, I pay tribute to a brilliant partner, a fun and level-headed son, a supportive extended family and the passionate, committed and industrious Desmond Tutu HIV Centre and Health Foundation family. I also remember in deep gratitude his life and teachings, our beloved patron, Archbishop Emeritus Desmond Mpilo Tutu, whom we lost on 29 December 2021. We proudly and diligently carry the baton he left with us forward.

